# Screening of Different Organs of Rats for HCA2 Receptor mRNA

**Published:** 2014

**Authors:** Tahoora Shomali, Najmeh Mosleh, Mohammad Kamalpour

**Affiliations:** 1 *Division of Pharmacology and Toxicology, Department of Basic Sciences, School of Veterinary Medicine, Shiraz University, Shiraz, Iran.*; 2 *Avian Disease Research Center, School of Veterinary Medicine, Shiraz University, Shiraz, Iran.*

**Keywords:** HCA2 receptor, mRNA, rat, RT-PCR

## Abstract

Interest in hydroxy - carboxylic acid 2 (HCA2) receptor has been raised since it is the target of antidyslipidemic drug nicotinic acid. The present study aimed to evaluate the presence of mRNA of this receptor in different organs of laboratory rat. Twenty two different organs of rats including mesenteric fat, epididymis (head, body and tail), testis, ovary, xiphoid process, liver, adrenal gland, femoral head, proximal epiphyseal and metaphyseal bone marrow of femur, esophagus, glandular stomach, forestomach, intestines, colons, heart, spleen, kidney, trachea, lung, skeletal muscle (quadriceps), cerebrum and cerebellum were removed and examined for HCA2 mRNA by RT- PCR method. The mRNA for HCA2 receptor was detected in all analyzed tissues. In conclusion, the different organs of rat express HCA2 receptor mRNA which makes a proper animal model for future studies on the physiological and pharmacological roles of this receptor *in vivo*.

The hydroxy - carboxylic acid (HCA) receptors including HCA1, HCA2 and HCA3 are a family of G protein-coupled receptors with high sequence homology (1). All of these receptors are predominantly expressed in adipose tissue (2) and have affinity for several intermediates of energy metabolism (1). HCA1 (GPR81) is endogenously activated by lactate, HCA2 (GPR109A) by 3-hydroxy-butyrate and HCA3 (GPR109B) by 3-hydroxylated β-oxidation intermediates, especially 3-hydroxy-octanoic acid (3-6). Of the HCA receptor family, HCA2 is the most extensively studied, since it is the target of the antidyslipidemic drug nicotinic acid (or niacin) (7-9). The physiological and pharmacological roles of HCA2 receptors are different according to the organ that has expressed the receptor. In adipose tissue, it is very likely that the HCA2 receptor is activated by 3-hydroxy-butyrate during starvation and mediates a negative feedback regulation that controls the rate of lipolysis (10) which can avoid excessive trigly-ceride degradation and thereby save energy during food shortage. Besides its antilipolytic effect, nicotinic acid (as a ligand for HCA2 receptor) has been shown to influence the function of the adipose tissue as an endocrine organ. Both *in vitro* and *in vivo* data indicate that nicotinic acid increases the release of adiponectin from adipocytes (11-12). On the other hand, nicotinic acid induces a biphasic increase in dermal blood flow which is mediated by HCA2 receptors. While the first phase is due to activation of HCA2 on Langerhans cells, the second phase of the flush depends on HCA2 expressed by keratinocytes (13). The nicotinic acid receptor HCA2 is also expressed by monocytes and macrophages including plaque macrophages, and nicotinic acid inhibits the recruitment of macrophages into atherosclerotic lesions in HCA2-dependent manner. In addition, HCA2 mediates a stimulatory effect of nicotinic acid on the cholesterol efflux from macrophages (14). There is evidence indicating that the HCA2 receptor expressed in intestinal epithelial cells responds to butyrate which is present in millimolar concentrations in the gut lumen, and that HCA2 thereby functions as a tumor suppressor and anti-inflammatory receptor (15). In 2009, Martin et al., demonstrated that HCA2 receptor is expressed in mammalian retinal pigment epithelium and may have a role in diabetic retinopathy (16).

Laboratory rats have served as an important animal model for research in different fields of medical sciences. Over the years, rats have been used as animal models in many experimental studies, which have expanded our knowledge in diverse aspects of biological sciences. In 2003, the human and rat HCA2 receptors were cloned and showed to be highly homologous to the murine orthologue PUMA-G (7-8). As far as we know, the distribution of HCA2 receptors in different organs of laboratory rats has not been addressed previously. Recognition of the organs that express this receptor may pave the road for future studies which may clarify new roles of HCA2 receptors that provides rationality for the present study.

## Materials and Methods

**Samples**

The samples from 22 different organs of 4 adult Sprague-Dawley rats were immediately removed after slaughtering them under deep anesthesia. The samples included: mesenteric fat, epididymis (head, body and tail), testis, ovary, xiphoid process, liver, adrenal gland, femoral head, proximal epiphyseal and metaphyseal bone marrow of femur, esophagus, glandular stomach, forestomach, intestines, colons, heart, spleen, kidney, trachea, lung, skeletal muscle (quadriceps), cerebrum and cerebellum. It should be mentioned that mesenteric fat, liver, bone marrow and spleen samples were obtained from both male and female rats, testis, epididymis, heart and adrenal gland from male rats and other samples from female rats. All samples were immediately moved to-70°C freezer until use.

The rats spent a week of adaptation period before sampling time, during which they had free access to chew pellets and tap water. The methods used in the present study are in accordance with institutional guideline of Shiraz University for care and use of laboratory animals.

**RT-PCR**

Same amount of samples from each organ were pooled for each analysis as described by Martin et al. (16). Except for mesenteric fat, which was frozen by liquid nitrogen and homogenized in a mortar; all samples were defrosted on ice and homogenized by an electric homogenizer. Eighty mg of pooled sample was used for RNA extraction. RNA was extracted by RNX^TM^ (-plus) (CinnaGen Co, Tehran, Iran) commercial kit according to manufacturer’s instructions. After DNase (Fermentas, Vilnius, Lithuania) treatment, ten µL of extracted RNA was reverse transcribed using AccuPower^®^-Rocket Script RT Pre Mix (Bioneer Co., Daejeon, South Korea) kit. F and R primer pair within the *Rat GPR109A (rPUMA-G) *gene was used with the following sequences: F: 5΄CTTTCT-GGTGATAAACGGCAAGA 3΄ and R: 5΄ GACT-GTCAGGCCGATGGTG 3΄ as described by Martin et al. (2009) for cDNA synthesis (16). Five μL of the cDNA was used for PCR amplificationusing Accu Power^®^-PCR Pre Mix with the above mentioned primer pair which yields in specific amplification of a 455bp fragment. 

The PCR thermocycling condition for the gene was as follows: Initial denaturation at 95°C for 3 min and then 35 cycles with denaturation at 95°C for 45 sec, primer annealing at 58°C for 30 sec and primer extension at 72°C for 30 sec and a final extension step at 72°C for 4 min. Five μL of PCR product was subjected to 1% agarose gel electrophoresis containing ethidium bromide and visualized under ultra-violet light.

**Fig. 1:**
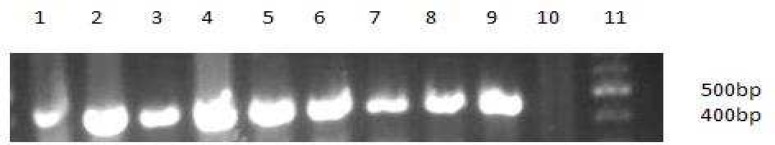
RT-PCR analysis of RNA collected from the different organs of rats. HCA2 receptor mRNA was detected in all samples. 1: cerebrum; 2: lung; 3: mesenteric fat; 4: skeletal muscle; 5: kidney; 6: ovary; 7: epididymis; 8: liver; 9: bone marrow; 10 negative control and 11: 100 bp ladder

## Results

Figure 1 shows the RT-PCR products of some organs included in the study after gel electro-phoresis. The mRNA for HCA2 receptor was found in all tissues analyzed.

## Discussion

The vast distribution of HCA2 mRNA in the different organs of rat, as observed in the present study, may make it a proper animal model for future investigations on physiological and pharmacological roles of this receptor in different organs and represents an opportunity for evaluation of the effects of pharmaceutical agents on expression of HCA2 receptors *in vivo*. It should be mentioned that the distribution pattern of HCA2 mRNA is different among species, for example Titgemeyer et al. (2011), reported that the liver of cattle expresses notably great amounts of HCA2 receptor mRNA (17), while Li et al. (2010) established that HCA2 is expressed in murine liver but its basal expression is low (18). In the present study, we found HCA2 receptor mRNA in liver sample of rat; however we cannot distinguish whether this mRNA was expressed by hepatocytes or by other cells especially Kupffer cells. This fact is also true for other organs examined in the present study, where it remains to be clarified exactly what type of cells have expressed the mRNA for HCA2 receptor. On the other hand, although we tried not to include fat in our samples from different organs, very low amount of fat may be present in our samples that can lead to RT-PCR amplification. Regarding the facts that all PCR reactions were performed according to the same protocol and on the same amount of samples and also that appreciable amounts of product was retrieved on gel; the chance for the implication of few adipocytes in this observation is low, although we cannot completely ignore it.

The presence of HCA2 receptor mRNA in cerebellum and 4 regions of cerebrum of cattle as well as skeletal muscle of this species has been previously described (17), which is consistent with the results of our study. Expression of HCA2 receptor gene in intestine and colon of mice and human has also been reported (19). As far as we know, this is the first time that expression of HCA2 in epididymis, testis, ovary, xiphoid process, adrenal gland, femoral head, proximal epiphyseal and metaphyseal bone marrow of femur, esophagus, glandular stomach, forestomach, heart, spleen, kidney, trachea and lung of laboratory rats has been demonstrated. Another drawback of our study is that we cannot conclude by our findings whether the presence of mRNA for HCA2 has led to expression of a functional receptor at protein level which remains to be confirmed in future studies.

In conclusion, this is a preliminary study that describes the presence of mRNA for HCA2 receptor in different organs of laboratory rat which makes it a proper animal model for future studies on the physiological and pharmacological roles of this receptor *in vivo*.
